# Impact of the Malnutrition on Mortality in Patients With Osteoporosis: A Cohort Study From NHANES 2005-2010

**DOI:** 10.3389/fnut.2022.868166

**Published:** 2022-05-11

**Authors:** Xiaohui Shangguan, Jialing Xiong, Shanshan Shi, Ying Liao, Liling Chen, Jiayi Deng, Wanxia Wu, Junjie Wang, Jiabin Tu, Jiaming Xiu, Weihao Wu, Longtian Chen, Kaihong Chen

**Affiliations:** ^1^Longyan First Affiliated Hospital of Fujian Medical University, Longyan, China; ^2^The Third Clinical Medical College, Fujian Medical University, Fuzhou, China

**Keywords:** malnutrition, mortality, osteoporosis, NHANES, prognosis

## Abstract

**Background:**

Osteoporosis is the most common metabolic bone disease. Recent studies have shown that malnutrition can promote the development of osteoporosis. However, the incidence of malnutrition in patients with osteoporosis and the relationship between malnutrition and all-cause mortality has not been adequately studied. Therefore, our study investigated the relationship between malnutrition and all-cause mortality in patients with osteoporosis.

**Methods:**

We analyzed data on 7,700 adults ≥20 years of age during National Health and Nutrition Examination Survey (NHANES) 2005-2010. Each patient was assigned to one of three groups: normal nutritional status, mild malnutrition, and moderate to severe malnutrition. Survival curves and univariate and multivariable cox regressions based on the NHANES recommended weights were used to assess the association between malnutrition status and mortality. Moreover, cox proportional hazards regression analyses were performed on the matched pairs.

**Results:**

Overall, 7,700 eligible individuals with osteoporosis were included in the final analysis, and the mean age was 52.0 ± 0.4 years. From the Kaplan–Meier curves for long-term all-cause mortality of malnutrition, worsening malnutrition status was associated with higher incidence of all-cause mortality. In the fully adjusted models, the adjusted hazard ratio (aHR) was 1.54 [95% confidence interval (CI), 1.02–2.31, *p* = 0.039] at mild malnutrition status and 2.70 (95%CI, 1.95–3.74, *p* < 0.001) at moderate to severe malnutrition status. The cox model after matching indicated that malnutrition was still a high mortality risk than no malnutrition (aHR = 2.23, 95% CI, 1.66–3.01, *p* < 0.001).

**Conclusions:**

Poor malnutrition status, common in osteoporotic patients, is strongly associated with a risk for all-cause mortality comparable to that seen with normal nutritional status. These findings highlight the importance of risk stratification for nutritional status in osteoporotic patients and the implementation of strategies that is now available to help prevent malnutrition in these patients.

## Introduction

Osteoporosis is the most common metabolic bone disease. It has a prevalence of 18.3% and affects approximately 1.2 billion people worldwide (1). It is characterized by low bone mass and microstructure changes that make bones vulnerable to fracture (2). Fracture is the most important consequence of osteoporosis and is associated with significant costs, morbidity, and mortality (3).

In recent studies, it has been shown that malnutrition can promote the development of osteoporosis (4), thereby further promoting the development of fractures (5). The advantage of malnutrition over other clinical variables is that it is a modifiable risk factor that physicians can act on (6). In the hospital setting, malnutrition is a highly prevalent condition, with 20–50% of patients malnourished at a single period of time (7). However, current evidence on the prognostic impact of malnutrition on bone disease is mainly focused on patients with fractures (8). The incidence of malnutrition in patients with osteoporosis and the relationship between malnutrition and all-cause mortality have not been adequately studied.

Therefore, our study investigated the relationship between malnutrition as determined by the nutritional risk Index (NRI) score and all-cause mortality in patients with osteoporosis by using the 2005-2010 National Health and Nutrition Examination Survey (NHANES).

## Methods

### Study Population

The NHANES is a nationally representative cross-sectional survey recursively conducted in the U.S. by the National Center for Health Statistics (NCHS) every 2 years, with 5,000 individuals sampled in each survey. The survey, at every survey period, obtains written informed consent from all participants and approval from the Ethics Review Board of the NCHS [Protocol #98e12; available on the web at NHANES-National Health and Nutrition Examination Survey Homepage (cdc.gov)].

**Figure 1:**
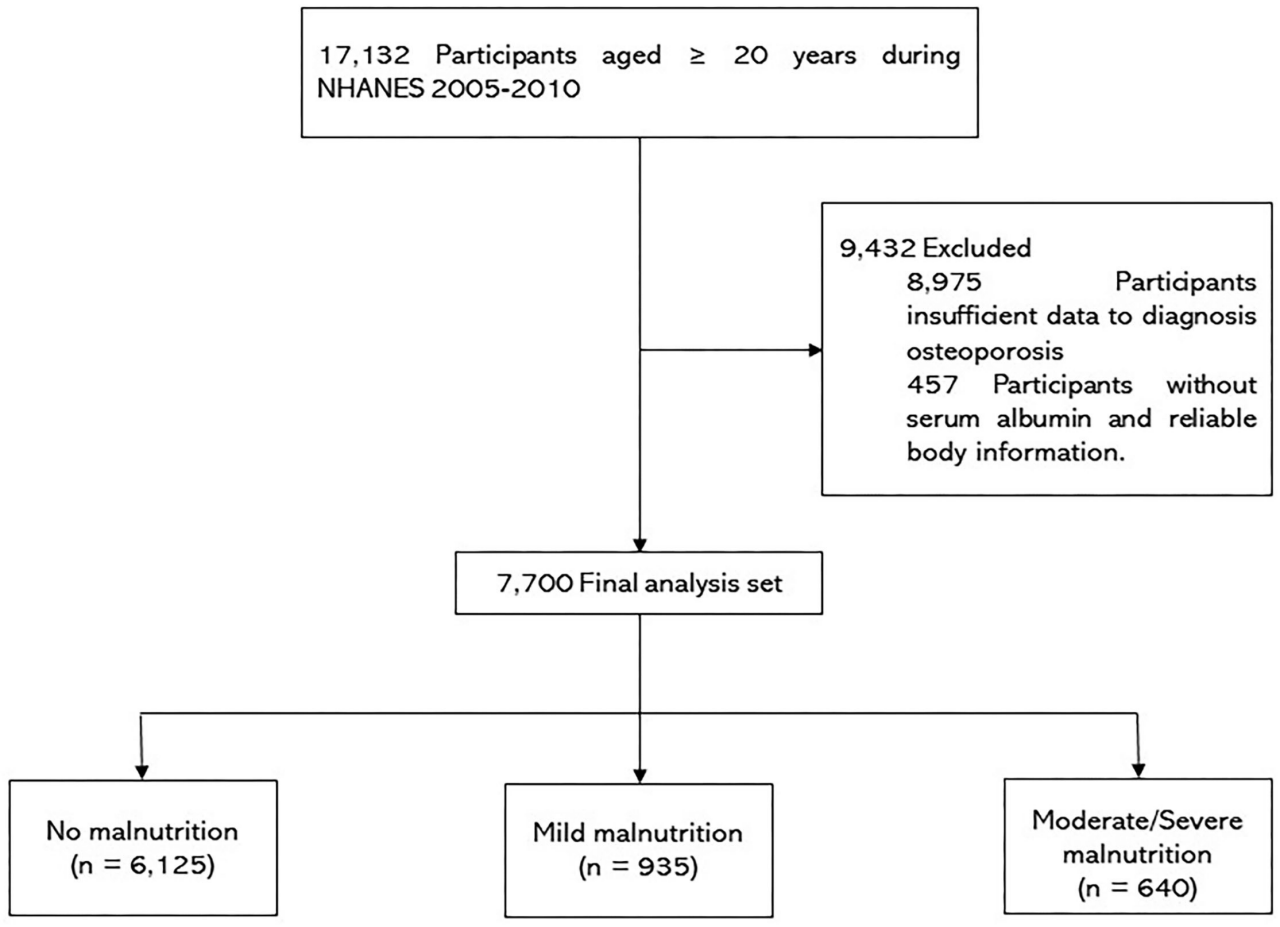
Flowchart.

As shown in Figure 1, this study included participants ≥ 20 years of age during NHANES 2005-2010 (*n* = 17,132). Of these participants, 9,432 were excluded based on the following: (i) insufficient data to diagnosis osteoporosis; and (ii) patients without serum albumin and reliable body information. Thus, 7,700 patients were enrolled in the present study.

### Definitions and Outcomes

The patient was diagnosed with osteoporosis based on the dual-energy X-ray absorptiometry (DXA)-determined bone mineral density (BMD) of the total femur, femoral neck, trochanter, intertrochanter, Ward's triangle, total spine, and vertebrae L1–L4. Patient diagnosis of osteoporosis was confirmed if one of three criteria was met: (1) femur neck BMD <0.558 g/cm^2^ (9); (2) T-score < −2.5 standard deviations (SDs); and (3) if the patient said “yes” to the question: “Has a doctor ever told you that you had osteoporosis, sometimes called ‘thin or brittle bones'?” [T-score = (BMD-reference BMD]) reference SD, reference group: 20- to 29-year-old non-Hispanic white women]. The NRI is a nutritional assessment score that has become popular in recent years due to its simplicity and strong prognostic value for different medical and surgical patient populations. It uses the following formula: 1.519 × serum albumin (g/l) + 41.7 × [current body weight (kg)/usual body weight (kg)] (10). NRI was first proposed by the Veterans Total Parenteral Nutrition Study Group (11) and consists of albumin, current weight, and usual weight, is objective, and easy to use compared to the aforementioned nutritional screening tools. It can quickly identify malnourished patients to better assess nutritional status. Therefore, the NRI was used to assess the nutritional status of patients with osteoporosis in order to analyze the prognosis of nutritional status on patients with osteoporosis in our study. According to previous study (12), patients were classified into 4 nutritional risk categories: severe nutritional risk (NRI <83.5), moderate nutritional risk (83.5 ≤ NRI <97.5), mild nutritional risk (97.5 ≤ NRI <100), and no nutritional risk (NRI ≥ 100), while the primary outcome was mortality. Mortality status and cause of death were determined by NHANES. We combined severe nutritional risk with moderate nutritional risk because the number of patients at severe nutritional risk was fewer. The National Death Index public-access files through December 31, 2015.

### Statistical Analysis

We used the NHANES-recommended weights to account for planned oversampling of specific groups. The continuous variables were expressed as the mean ± standard deviation, and the categorical variables were presented as counts (percentages). Baseline characteristics between the three malnutrition groups were compared using an ANOVA for continuous variables and an χ*2* test for categorical variables.

Each patient was assigned to one of three groups: normal nutritional status, mild malnutrition, and moderate to severe malnutrition. To evaluate the association between different malnutrition status and mortality, we used Kaplan Meier estimates to calculate cumulative survival probabilities for all-cause mortality and univariate and multivariate cox regression analyses. Both the estimates and probabilities are based on the NHANES-recommended weights. Afterwards, hazard ratio (HR) and 95% confidence interval (CI) were calculated. Model 1 was a crude model unadjusted for potential confounders. Model 2 was adjusted for demographic factors, including age, sex, and race/ethnicity. Model 3 was further adjusted for body mass index (BMI), congestive heart failure (CHF), diabetes mellitus (DM), hypertension, and cancer. Restricted cubic splines were used to evaluate linear and non-linear associations. To demonstrate the reliability of the results, we matched the basic characteristics of the normal nutritional and malnutrition groups using propensity score matching. Moreover, cox proportional hazards regression analyses were performed on the matched pairs. We further explored the relationship between different malnutrition status and mortality in different subgroups (age, sex, race/ethnicity, CHF, DM, hypertension, and cancer). All data analyses were performed using Survey package in R software (version 4.0.4; R Foundation for Statistical Computing, Vienna, Austria).

## Results

### Clinical Characteristics

Overall, 7,700 eligible individuals with osteoporosis were selected from the publicly available NHANES and included in the final analysis. Their mean age was 52.0 ± 0.4 years. There were 269 (2.6%) patients with CHF, 897 (7.8%) patients with DM, 2,895 (32.2%) patients with hypertension, and 946 (11.9%) patients with cancer. The average BMI of the population was 26.9 ± 0.1 kg/m^2^. Most of the patients were female (58.6%), and 76.4% were of the Non-Hispanic White. According to the NRI scoring criteria, 7,700 patients were divided into three groups: normal nutritional status, mild malnutrition, and moderate to severe malnutrition. Nine hundred thirty-five patients suffered from mild malnutrition, while 640 patients had moderate to severe malnutrition. Patients with malnutrition were older and were more likely to be female. Compared with those with normal nutritional status, the higher the level of malnutrition, the higher the incidence of CHF, DM, hypertension, and cancer. More data on the baseline characteristics of study population are shown in Table 1. Propensity score matching yielded 3,090 patients of similar populations (i.e., no statistically significant differences) in the matched cohort for most variables (Supplementary Table 1).

**Table 1:** Baseline characteristics of the study population (weighted).

**Characteristics**	**Total** **(*n* = 7,700)**	**No malnutrition** **(*n* = 6,125)**	**Mild malnutrition** **(*n* = 935)**	**Moderate/severe malnutrition** **(*n* = 640)**	***p*** **-value**
Age	52.0 ± 0.4	51.2 ± 0.4	54.6 ± 0.9	57.7 ± 0.8	<0.001
Female	4,342 (58.6)	3,263 (55.1)	659 (76.1)	420 (72.9)	<0.001
**Race/ethnicity**					
Mexican American	1,356 (6.9)	1,128 (7.0)	139 (5.9)	89 (5.4)	<0.001
Non-Hispanic white	4,342 (76.4)	3,491 (77.0)	507 (73.3)	344 (74.4)	
Non-Hispanic black	965 (6.2)	668 (5.3)	164 (9.8)	133 (11.1)	
Other	1,037 (10.6)	838 (10.7)	125 (11.1)	74 (8.7)	
**Education**					
<12	4,100 (53.32)	3,211 (42.3)	510 (44.5)	379 (50.1)	0.012
12	2,009 (26.01)	1,599 (29.1)	248 (30.6)	162 (28.6)	
>12	1,576 (20.47)	1,302 (28.7)	176 (24.9)	98 (21.4)	
BMI	26.9 ± 0.1	26.8 ± 0.1	28.0 ± 0.3	26.1 ± 0.4	<0.001
CHF	269 (2.6)	176 (2.1)	46 (3.8)	47 (6.4)	<0.001
DM	897 (7.8)	660 (7.1)	120 (10.7)	117 (12.6)	0.003
Hypertension	2,895 (32.2)	2,222 (31.2)	391 (36.3)	282 (38.7)	0.002
Cancer	946 (11.9)	695 (11.0)	136 (14.6)	115 (17.8)	<0.001

*BMI, body mass index; CHF, congestive heart failure; DM, diabetes mellitus*.

### Primary Outcomes

**Figure 2:**
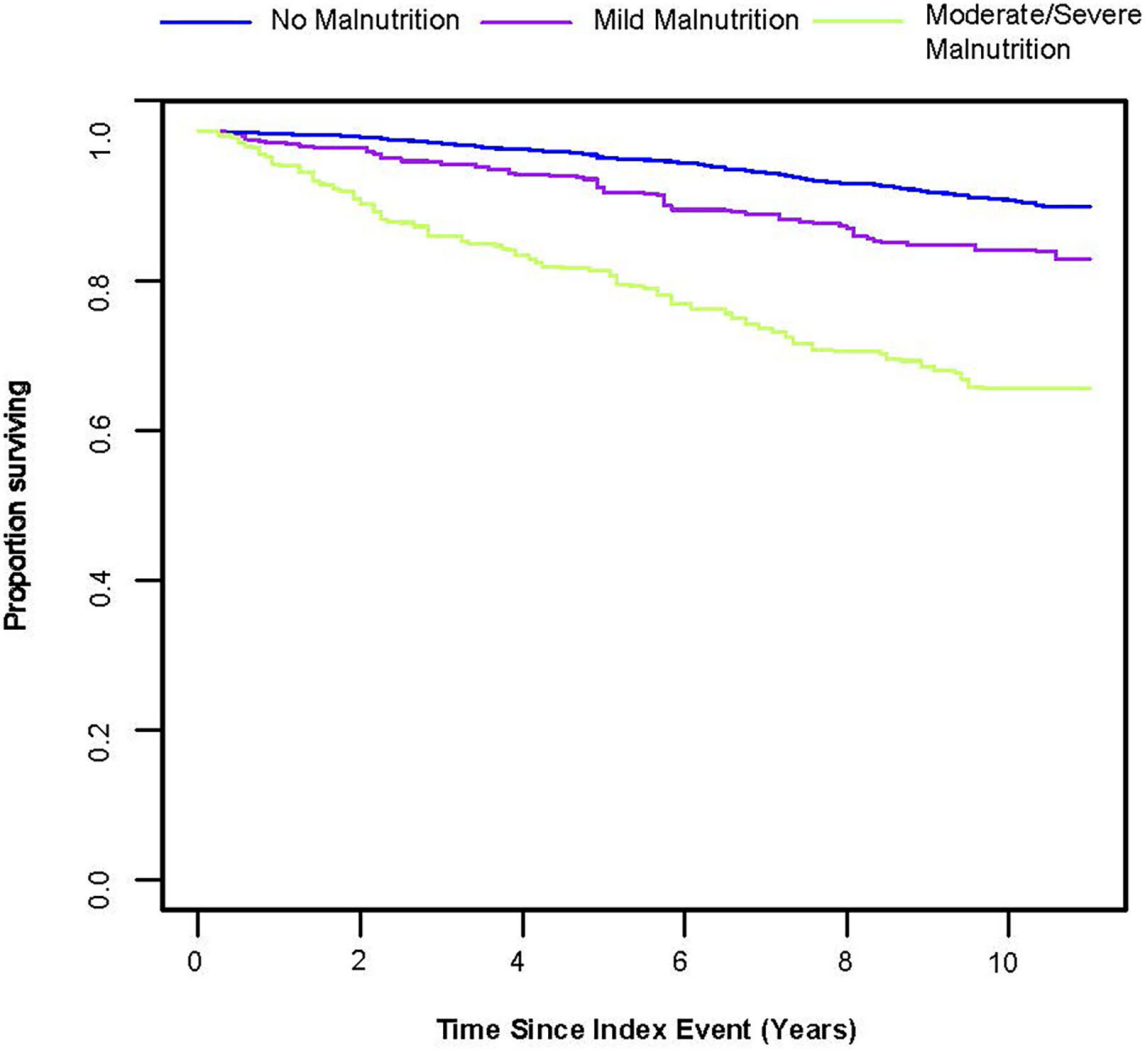
Kaplan-Meier Survival Estimates for long-term all-cause mortality (weighted).

**Figure 3:**
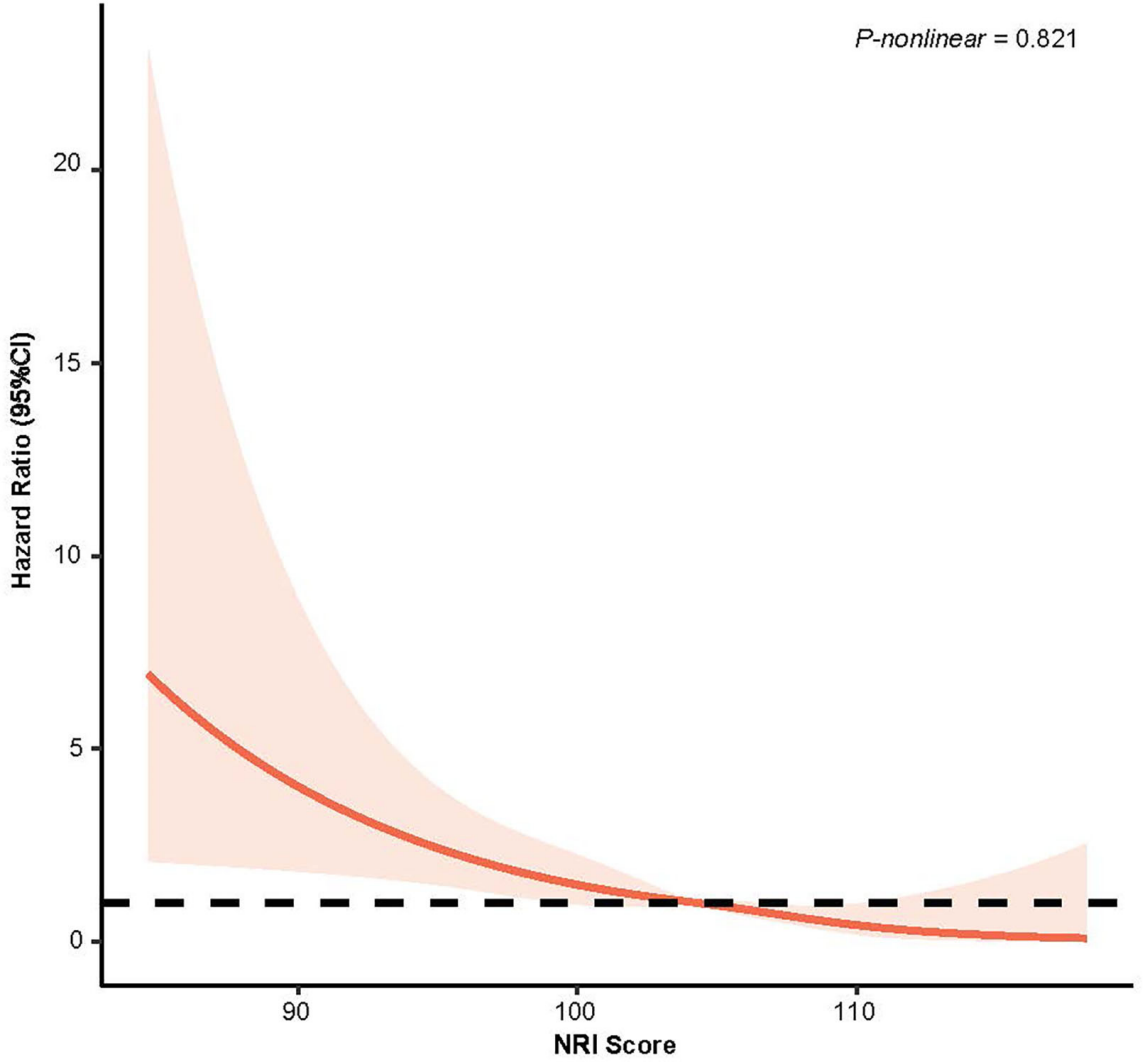
Restricted Cubic Splines of Hazard Ratio (HR) and 95% CI for the association between NRI score (continuous) and all-cause mortality (unweighted).

From the Kaplan–Meier curves for long-term all-cause mortality of malnutrition, worsening malnutrition status had higher all-cause mortality (Figure 2). The Cox proportional hazards regression analysis indicated that compared with normal nutritional status, malnutrition was associated with significantly increased risk for long-term all-cause mortality. At the unadjusted model 1 and adjusting for components of model 2, malnutrition was associated with significantly increased risk for all-cause mortality [HR for moderate to severe degrees of malnutrition, respectively: 4.35 (95% CI, 3.04 to 6.23) for unadjusted model 1, 3.07 (95% CI, 2.25 to 4.20) for the model 2; *p* < 0.001 for all models]. In the fully adjusted models, the adjusted hazard ratios (aHR) were 1.54 (95%CI, 1.02–2.31, *p* = 0.039) at mild malnutrition status and 2.70 (95%CI, 1.95–3.74, *p* < 0.001) at moderate to severe malnutrition status (Table 2). After matching, the cox model indicated that malnutrition was still a high mortality risk compared to no malnutrition (aHR = 2.23, 95% CI, 1.66–3.01, *p* < 0.001, Supplementary Table 2). Restricted cubic spline analysis confirmed a strong linear association between NRI score and HR for patients with osteoporosis (*P-nonlinear* = 0.821; Figure 3).

**Table 2:** All-cause mortality hazard ratios (HRs) for participants aged 20 years and older according to malnutrition status.

**Status**	**Model 1**		**Model 2**		**Model 3**	
	**HR (95%CI)**	***P*** **-value**	**HR (95%CI)**	***P*** **-value**	**HR (95%CI)**	***P*** **-value**
**Continuous**						
NRI (per 1 score)	0.90 (0.87–0.93)	<0.001	0.91 (0.88–0.95)	<0.001	0.92 (0.89–0.95)	<0.001
**Categories**						
No malnutrition	1 [Ref]	NA	1 [Ref]	NA	1 [Ref]	NA
Mild malnutrition	1.79 (1.21–2.67)	0.004	1.47 (0.96–2.27)	0.079	1.54 (1.02–2.31)	0.039
Moderate/severe malnutrition	4.35 (3.04–6.23)	<0.001	3.07 (2.25–4.20)	<0.001	2.70 (1.95–3.74)	<0.001

*National Health and Nutrition Examination Survey (NHANES) 2005 to 2010 with follow-up through 2015. Model 1: No adjusted. Model 2: Adjusted by age, gender, race/ethnicity. Model 3: Adjusted by age, gender, race/ethnicity, BMI, CHF, DM, hypertension, Cancer*.

### Subgroups Analyses

**Figure 4:**
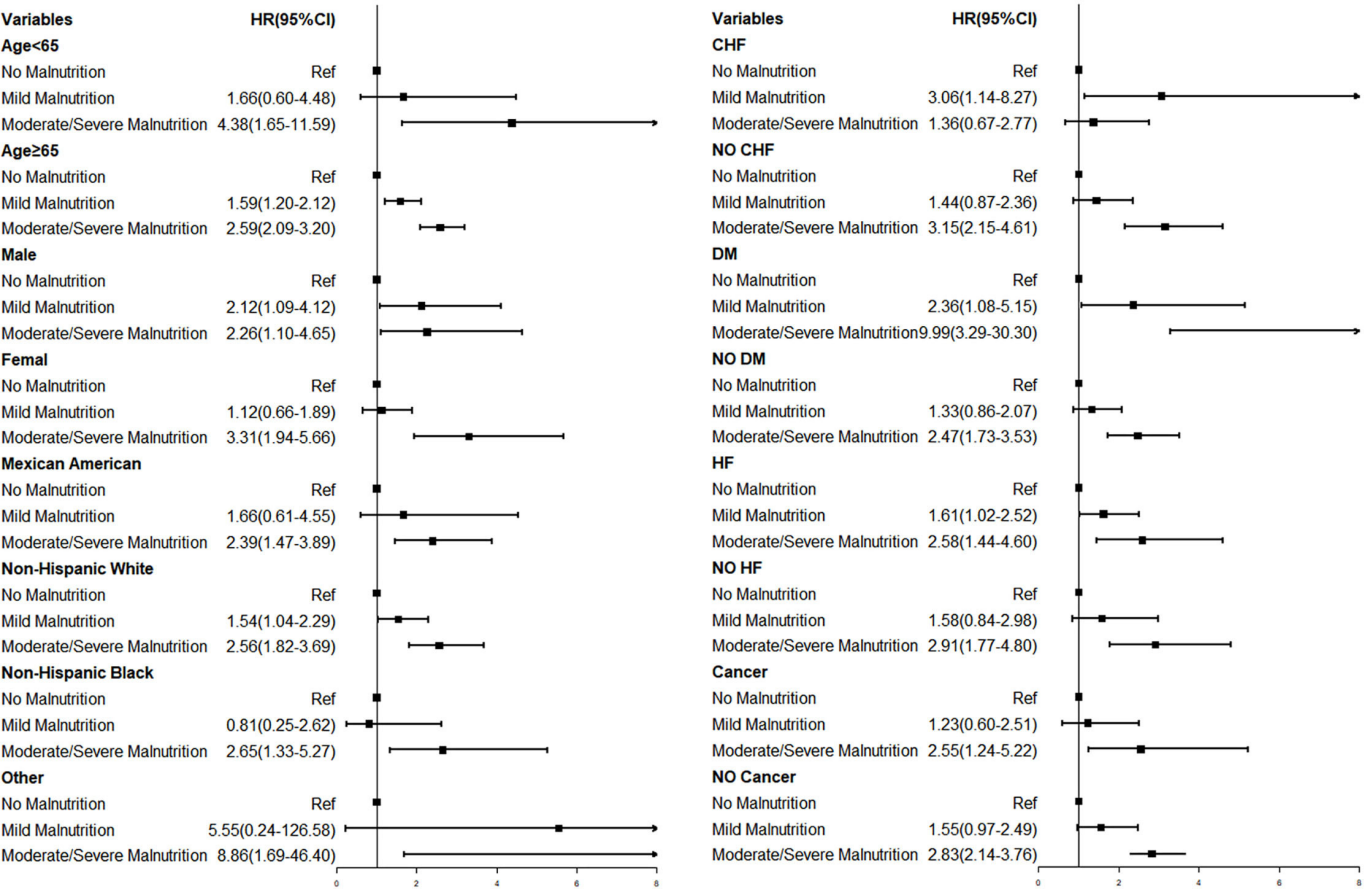
Forest plot of overall survival in subgroups. CI, confidence interval; HR, Hazard ratio.

Subgroup analysis showed that in most subgroups, the malnutrition group was associated with a higher risk of mortality (Figure 4). For the elder patient, malnutrition increased the risk of mortality in both severities (Mild Malnutrition: HR = 1.59, 95% CI, 1.20–2.12, *p* = 0.001; Moderate/Severe Malnutrition: HR = 2.59, 95% CI, 2.09–3.20, *p* < 0.001). In addition, in the diabetes subgroup, moderate to severe malnutrition increased the risk of mortality by 899% (*p* < 0.001).

## Discussion

As far as we know, this is the first study to examine the mortality risk of malnutrition in the population of patients with osteoporosis. In this study, malnourished patients with osteoporosis accounted for nearly one-fifth of the included population according to NRI score. Our study reported that the malnourished osteoporotic population has a higher all-cause mortality risk compared to the osteoporotic population with normal nutritional status.

Malnutrition may affect the prognosis of patients with osteoporosis through major mechanisms. Malnutrition is a complex state that includes a decrease in protein reserves and a collapse in calories which can weaken immune defenses. Decreased immune defenses are a key factor in the development and progression of many chronic diseases (13). An increase in inflammatory cytokines is a hallmark of inflammatory chronic diseases. Chronic inflammation is often associated with physical exertion which can over time lead to malnutrition. In addition, certain inflammatory factors, such as interleukin 6, have a detrimental effect on bone tissue. Interleukin-6 increases osteoclast activation, leading to impaired local and/or systemic growth hormone/insulin-like growth factor 1 signaling (4). Persistent inflammation is an important driver of disease progression as it affects prognosis (14). Therefore, it is reasonable to assume that inflammation contributes to high mortality risk in patients with osteoporosis.

There were some interesting results. In the age subgroup of patients with osteoporosis, patients older than 65 years who developed mild malnutrition was also significantly associated with a higher risk of mortality. It was found that in elderly people older than 65 years old, bone density is susceptible to nutritional status, which in turn indicates that malnourished elderly patients are prone to osteoporosis. Once a fracture occurs, complications, such as blood hypercoagulation, complicating embolism, and lung infection due to slow bone recovery in old age, and prolonged bed rest, will lead to the death of the patient (15). In addition, with the increase of age, the intestinal absorption capacity gradually decreases, and the lack of calcium will lead to bone resorption, aggravating the poor prognosis of patients with fractures. Moreover, our study showed that moderate to severe malnutrition had an even greater impact on the mortality of patients with osteoporosis in the <65 years subgroup compared with the elderly population. Therefore, we should pay equal attention to these patients. In addition, clinical exams found that osteoporosis has a clear gender tendency. Results of gender subgroups show that moderate to severe malnutrition has a greater impact on female patients with osteoporosis compared with males. In addition, bone synthesis in females is closely related to endocrine function. Therefore, malnourishment affects the regulation of hypothalamic-pituitary-ovarian axis function and its related target cell effects, leading to the disruption of hormone levels in the body and aggravating the progression of osteoporosis. Recent studies found that DM was an important risk factor for osteoporosis, and that the risk and incidence of fractures are higher in diabetic patients compared to non-diabetic patients (16, 17). Our study had also concluded similar results. Diabetic patients had a higher risk of all-cause mortality in patients with osteoporosis, regardless of their degree of malnutrition.

Exercises and medications have an important role in the prevention and treatment of patients with osteoporosis (18, 19). In addition to medication and exercise management, early nutritional intervention and strengthening nutritional management are also important for the prognosis of patients with osteoporosis. Nutrition is an important factor in the prognosis of many human diseases. In line with this, the premise of nutritional intervention is to detect malnutrition in a timely manner so as to reverse the trend of malnutrition and improve the poor prognosis of patients with osteoporosis. Moreover, the nutritional intervention is never simply the supplementation of a single factor or, at times, over-supplementation, but is a reasonable dietary mix according to individual needs to reduce the risk of osteoporosis and mortality. Therefore, clinicians need to pay attention to malnutrition detection to reverse its trend and formulate reasonable countermeasures for individualized interventions.

This study has several limitations. First, our outcome event was restricted to all-cause mortality, but it was an unbiased and clinically relevant outcome. In addition, the sample size of our study was large enough to demonstrate a strong association between malnutrition and osteoporosis. Second, our study was a retrospective analysis that used data from the 2005-2010 NHANES, which may have poor timeliness. Third, only one assessment method, NRI, was used to assess the malnutrition level of patients. We used a single evaluation method, which has some limitations. Hence, data may be biased. However, NRI has higher objectivity and accuracy compared with other evaluation methods.

## Conclusion

Our study discussed the effect of different nutritional status on the prognosis of patients with osteoporosis and found that the poor malnutrition status common in these patients is strongly associated with a risk for all-cause mortality that is comparable to that seen in normal nutritional status. These findings highlight the importance of risk stratification for nutritional status in osteoporotic patients and the implementation of strategies that are now available to help prevent malnutrition in these patients.

## Data Availability Statement

Publicly available datasets were analyzed in this study. This data can be found at: https://www.cdc.gov/nchs/nhanes/.

## Ethics Statement

The survey obtains written informed consent from all participants and approval from the Ethics Review Board of the NCHS. Written informed consent for participation was not required for this study in accordance with the national legislation and the institutional requirements.

## Author Contributions

XHSG participated in formulating the research question, design of analyses, interpretation of the data, drafting the manuscript, revising the manuscript, and the approval of the final version. JLX participated in the design of analyses, revision of the manuscript, and approved the final version. SSS contributed to the interpretation of the data, drafting the manuscript, revision of the manuscript, and the approval of the final version. YL, LLC, JYD, JJW, JBT, and JMX revised the manuscript and approved the final version. WHW participated in formulating the research question, design of analyses, the revision the manuscript, and approved the final version. LTC participated in formulating the research question, design of analyses, revision of the manuscript, and approved the final version. KHC participated in formulating the research question, design of analyses, data analysis, interpretation of the data, and approved the final version. All authors read and approved the final version of the manuscript and are responsible for all aspects of the manuscript.

## Funding

This research was funded and supported by the Young and Middle-aged Expert Fund for Outstanding Contributions to Hygiene and Health in Fujian Province.

## Conflict of Interest

The authors declare that the research was conducted in the absence of any commercial or financial relationships that could be construed as a potential conflict of interest.

## Publisher's Note

All claims expressed in this article are solely those of the authors and do not necessarily represent those of their affiliated organizations, or those of the publisher, the editors and the reviewers. Any product that may be evaluated in this article, or claim that may be made by its manufacturer, is not guaranteed or endorsed by the publisher.
