# Supplementary material for: Impact of the Malnutrition on Mortality in Patients With Osteoporosis: A Cohort Study From NHANES 2005-2010
Source: Front Nutr. 2022 May 11;9:868166. doi: 10.3389/fnut.2022.868166 (PMC9132007; doi:10.3389/fnut.2022.868166)
Supplement: Supplementary file 1 [file Table_1.DOCX]

**eTable 1 Baseline characteristics of the study population after propensity score matching (weighted).**

| **Characteristics** |  | **No Malnutrition**  **(n=1,545)** | **Malnutrition**  **(n=15,45)** |  | **p-value** |
| --- | --- | --- | --- | --- | --- |
| **Age** |  | 55.6 ± 0.6 | 55.6 ± 0.6 |  | 0.778 |
| **Female** |  | 1052 (71.3) | 1057 (74.7) |  | 0.859 |
| **Race/ethnicity** | | | | | |
| Mexican American |  | 193 (4.1) | 224 (5.7) |  | < 0.001 |
| Non-Hispanic White |  | 836 (79.0) | 943(73.8) |  |  |
| Non-Hispanic Black |  | 289 (6.1) | 204 (10.3) |  |  |
| Other |  | 196 (10.8) | 205 (10.2) |  |  |
| **Education** | | | | | |
| < 12 |  | 819 (43.4) | 867 (46.5) |  | 0.055 |
| 12 |  | 382 (27.0) | 403 (29.7) |  |  |
| > 12 |  | 341 (29.5) | 273 (23.8) |  |  |
| **BMI** |  | 26.8 ± 0.2 | 27.2 ± 0.2 |  | 0.949 |
| **CHF** |  | 75 (3.6) | 78 (4.2) |  | 0.992 |
| **DM** |  | 230 (10.9) | 222 (10.9) |  | 0.992 |
| **Hypertension** |  | 662 (36.8) | 651 (36.8) |  | 0.978 |
| **Cancer** |  | 243 (16.7) | 242 (15.6) |  | 0.534 |

**Abbreviation:** BMI, body mass index; CHF, congestive heart failure; DM, diabetes mellitus.
