# Supplementary material for: Impact of the Malnutrition on Mortality in Patients With Osteoporosis: A Cohort Study From NHANES 2005-2010
Source: Front Nutr. 2022 May 11;9:868166. doi: 10.3389/fnut.2022.868166 (PMC9132007; doi:10.3389/fnut.2022.868166)
Supplement: Supplementary file 2 [file Table_2.DOCX]

**eTable 2. All-cause mortality hazard ratios (HRs) for participants aged 20 years and older according to malnutrition status after propensity score matching: NHANES survey 2005 to 2010 with follow-up through 2015.**

| **Status** | **Model 1** | | **Model 2** | | **Model 3** | |
| --- | --- | --- | --- | --- | --- | --- |
|  | HR (95%CI) | P-value | HR (95%CI) | P-value | HR (95%CI) | P-value |
| **Continuous** | | | | | | |
| NRI (per 1 score) | 0.91 (0.88-0.93) | <0.001 | 0.90 (0.88-0.93) | <0.001 | 0.90 (0.89-0.92) | <0.001 |
| **Categories** | | | | | | |
| No Malnutrition | 1 [Ref] | NA | 1 [Ref] | NA | 1 [Ref] | NA |
| Mild Malnutrition | 2.10 (1.44-3.06) | <0.001 | 2.15 (1.49-3.11) | <0.001 | 2.23 (1.66-3.01) | <0.001 |

Model 1: No adjusted.

Model 2: Adjusted by age, gender, race/ethnicity.

Model 3: Adjusted by age, gender, race/ethnicity, BMI, CHF, DM, hypertension, Cancer.
